# The evolution in melanoma treatment as a reflection of precision-oriented medicine

**DOI:** 10.3892/ol.2012.1065

**Published:** 2012-12-05

**Authors:** IGAL KUSHNIR, OFER MERIMSKY

**Affiliations:** Department of Oncology, Tel Aviv Sourasky Medical Center, Tel Aviv 64239, Israel

**Keywords:** melanoma, treatment, precision medicine, BRAF inhibitor

## Abstract

Until recently, metastatic melanoma was a disease with limited treatment options and a poor prognosis. Dacarbazine was accepted as the standard treatment for melanoma in the 1970s, and despite inducing an overall survival of approximately 7.4 months, it remained so until relatively recently. In the last few years, significant advances in the molecular understanding of this disease have facilitated the development of novel and promising drugs. Precision-oriented medicine is currently revolutionizing the practice of oncology. Targeted therapies have demonstrated great potential in treating melanoma and various other types of cancer, including breast, colorectal and non-small cell lung cancer. Here, we review the evolution of melanoma treatment from single-agent chemotherapy to combination therapy, the emergence of immunotherapy in melanoma and the development of targeted therapies, such as the use of the BRAF inhibitor as a treatment agent. The ability to treat melanoma according to the fingerprint of the tumor reflects an overall change in the practice of oncology.

## Contents

1. Introduction
2. The evolution of chemotherapy
3. The evolution of immunotherapy
4. The era of precision
5. Conclusion

## 1. Introduction

Metastatic melanoma is a fatal disease with a poor prognosis. Melanoma of the skin is the fifth leading cancer by incidence in men and the seventh in women in the USA. The lifetime probability of developing melanoma of the skin for Caucasians in the United States is 2.67% (1 in 37) for men and 1.79% (1 in 56) for women. It was estimated that in the year 2010, 8700 patients in the United States will die from melanoma (1). The rising incidence of this fatal disease is quite alarming, as while remarkable advances have been made in other fields of oncology, the treatment of metastatic melanoma has seen almost no change in the last few decades (2) In recent years, however, significant progress has been made in the understanding of the disease, and the introduction of new agents has facilitated improvements in the treatment of melanoma. In this review, we describe the evolution of melanoma treatment, from different cytotoxic agents and general immunomodulators, towards precision-oriented medicine.

## 2. The evolution of chemotherapy

Until the acceptance of dacarbazine as a gold standard in the 1970s (3,4), the suitability of numerous cytotoxic agents in treating melanoma were investigated. Alkylating agents, in particular cyclophosphamide, had a modest effect. Antimetabolite agents, including 5-fluorouracil, methotrexate and 6-mercaptopurin, were even less effective. Vinca alkaloids and other agents such as mitomycin c, bleomycin and lomustine, were also investigated (3). As single-agent dacarbazine had achieved an overall survival of only 7.4 months and a response rate (complete and partial) of only 16.9%, combination therapy with and without dacarbazine was subsequently investigated (5).

Numerous combination treatments failed to demonstrate any advantage over single-agent dacarbazine. This can be partially explained by the fact that combinations were developed empirically and were not based on *in vitro* synergistic trials. Two combinations are worth mentioning: the CVD (cisplatin, vinblastin and dacarbazine) and the Dartmouth (cisplatin, carmustine, dacarbazine and tamoxifen) regimens. The CVD regimen appeared to have potential following a phase II trial, in which the overall response rate was 40% and the overall median survival time was 9 months. However, with a response rate of 13.8% in a phase III trial, which compared CVD and biochemotherapy, the CVD regimen appeared to be a less promising treatment (6,7). The Dartmouth regimen, which also demonstrated a high response rate in a phase II trial, was revealed to have a response rate of 18.5% (compared with 10.2% for single-agent dacarbazine) in a phase III trial. This increase in the response rate, however, was not statistically significant. In addition, no difference was observed in overall survival between the Dartmouth regimen and single-agent dacarbazine. Moreover, the toxicity rate of the Dartmouth regimen was higher than that of single-agent dacarbazine (8).

## 3. The evolution of immunotherapy

While searching for improved cytotoxic agents, it was recognized that the host immune system had the potential to be utilized in treating tumor cells. It was observed that malignant melanoma is capable of going through spontaneous regression, typically associated with lymphocytic infiltration, in which both cellular and humeral components were implicated (9,10). In addition, the association between vitiligo, which is an autoimmune disorder, and melanoma was examined. It was demonstrated that exposing melanoma cells to the sera from vitiligo patients inhibited tumor cells (11–13). Therefore, as it became evident that melanoma is an immunogenic tumor, agents that are general immune system stimulators were investigated. Progress was made when interleukin-2 (IL-2), a T-cell growth factor, was identified in 1976. IL-2 induced a response rate of approximately 16% in metastatic melanoma, which is little higher than that of dacarbazine. However, IL-2 was capable of inducing a durable, complete response in a greater number of metastatic patients than had been previously achieved. However, the high toxicity profile of IL-2 prevented its widespread use (14,15).

Ipilimumab, a novel immunotherapy agent, is an antibody directed against the cytotoxic T-lymphocyte associated antigen 4 (CTLA4) molecule expressed on lymphocytes. Blocking this receptor enhances the antitumor T-cell response. A phase III study compared treatment with ipilimumab, both with and without a peptide vaccine (glycoprotein 100), to treatment with glycoprotein 100 alone, in 675 HLA-A*0201 positive patients with stage III unresectable or stage IV melanoma. The median overall survival of patients receiving ipilimumab in combination with the vaccine was 10.1 months and was significantly greater than that of patients who had received the peptide vaccine alone (6.4 months; P=0.003). Thus, ipilimumab has become the new standard in immunotherapy (16).

## 4. The era of precision

During the 21st century, the era of precision-oriented medicine has revolutionized the practice of oncology. Rather than using general cytotoxic agents with considerable side effects due to the fact that they affect normal/healthy tissue, small molecules with specific targets have become a more popular mode of treatment. Their use is found in the treatment of breast, colorectal and non-small cell lung cancer (for example as trastuzumab, cetuximab and erlotinib, respectively), and currently in melanoma treatment. Significantly, the identification of mutations that enable the tumor cells to survive and proliferate uncontrollably underlie such advances in the evolution of cancer treatment. Molecular therapy is able to target such mutations.

In 2002, researchers at the Sanger Institute (Cambridge, UK) discovered that mutations in the gene encoding the serine-threonine protein kinase rapidly accelerated fibrosarcoma isoform B (BRAF) occurred in >60% of melanomas initially tested. Melanomas carrying a BRAF mutation constitutively activate the mitogen-activated protein kinase (MAPK) pathway, promoting cellular proliferation and preventing apoptosis (17). In a phase II study that set out to investigate a selective BRAF inhibitor, vemurafenib, in patients with metastatic melanoma, 6% had a complete response and 47% had a partial response to the agent (18). The BRIM 3 study, a phase III randomized clinical trial, compared vemurafenib with dacarbazine in 675 patients with previously untreated, metastatic melanoma with BRAF mutation. Vemurafenib was associated with a 63% decrease in the risk of mortality and a 74% decrease in the risk of either mortality or disease progression, relative to dacarbazine (P<0.001 for both comparisons). The response rates were 48% for vemurafenib and 5% for dacarbazine (19). These results led to the adoption of vemurafenib as the first-line agent in treating metastatic melanoma with BRAF mutation. BRAF mutations were also identified in other cancer cell lines, including gliomas, sarcomas, and lung and colon cancer (17). Further research needs to be conducted to evaluate the role of BRAF inhibitors in cancers other than melanoma.

Another possible targeted treatment for patients with metastatic melanoma is available for melanoma cells with a KIT mutation. In a study conducted by Curtin *et al*, KIT mutations or copy number increases were found in 39% of mucosal melanoma, 36% of acral melanoma and 28% of melanoma on skin with chronic sun-induced damage (20). Imatinib, a drug that became the standard for treating patients with gastrointestinal stromal tumors (GIST), may be effective in treating such melanomas (21).

## 5. Conclusion

Targeting critical mutated pathways has revolutionized the practice of oncology. As we have observed, little progress was made in treating metastatic melanoma until the last few years. There has been a progression from treating according to histology (using general cytotoxic agents), towards treating according to the genetic fingerprint of each tumor and matching its specific therapy. As previously discussed, this change in practice has significantly impacted the prognosis of melanoma, as well as that of other types of cancer, including breast, colorectal and non-small cell lung cancer. We hypothesize that by combining different molecular targets and therefore preventing the tumor cells from escaping, the idea that metastatic cancer is incurable may eventually change.

## References

[1] Jemal A, Siegel R, Xu J, Ward E (2010). Cancer statistics, 2010. CA Cancer J Clin.

[2] Hayat MJ, Howlader N, Reichman ME, Edwards BK (2007). Cancer statistics, trends, and multiple primary cancer analyses from the Surveillance, Epidemiology, and End Results (SEER) Program. Oncologist.

[3] Luce JK (1972). Chemotherapy of malignant melanoma. Cancer.

[4] Benjamin RS (1979). Chemotherapy of malignant melanoma. World J Surg.

[5] Huncharek M, Caubet JF, McGarry R (2001). Single-agent DTIC versus combination chemotherapy with or without immunotherapy in metastatic melanoma: a meta-analysis of 3273 patients from 20 randomized trials. Melanoma Res.

[6] Legha SS, Ring S, Papadopoulos N, Plager C, Benjamin R (1989). A prospective evaluation of a triple-drug regimen containing cisplatin, vinblastine, and dacarbazine (CVD) for metastatic melanoma. Cancer.

[7] Atkins MB, Hsu J, Lee S (2008). Phase III trial comparing concurrent biochemotherapy with cisplatin, vinblastine, dacarbazine, interleukin-2, and interferon alfa-2b with cisplatin, vinblastine, and dacarbazine alone in patients with metastatic malignant melanoma (E3695): a trial coordinated by the Eastern Cooperative Oncology Group. J Clin Oncol.

[8] Chapman PB, Einhorn LH, Meyers ML (1999). Phase III multi-center randomized trial of the Dartmouth regimen versus dacarbazine in patients with metastatic melanoma. J Clin Oncol.

[9] Lewis MG (1967). Possible immunological factors in human malignant melanoma in Uganda. Lancet.

[10] Tefany FJ, Barnetson RS, Halliday GM, McCarthy SW, McCarthy WH (1991). Immunocytochemical analysis of the cellular infiltrate in primary regressing and non-regressing malignant melanoma. J Inves Dermatol.

[11] Ram M, Shoenfeld Y (2007). Harnessing autoimmunity (vitiligo) to treat melanoma: a myth or reality?. Ann N Y Acad Sci.

[12] Merimsky O, Shoenfeld Y, Fishman P (1998). The clinical significance of antityrosinase antibodies in melanoma and related hypopigmentary lesions. Clin Rev Allergy Immunol.

[13] Fishman P, Azizi E, Shoenfeld Y (1993). Vitiligo autoantibodies are effective against melanoma. Cancer.

[14] Atkins MB, Lotze MT, Dutcher JP (1999). High-dose recombinant interleukin 2 therapy for patients with metastatic melanoma: analysis of 270 patients treated between 1985 and 1993. J Clin Oncol.

[15] Komenaka I, Hoerig H, Kaufman HL (2004). Immunotherapy for melanoma. Clin Dermatol.

[16] Hodi FS, O’Day SJ, McDermott DF (2010). Improved survival with ipilimumab in patients with metastatic melanoma. N Engl J Med.

[17] Davies H, Bignell GR, Cox C (2002). Mutations of the BRAF gene in human cancer. Nature.

[18] Sosman JA, Kim KB, Schuchter L (2012). Survival in BRAF V600-mutant advanced melanoma treated with vemurafenib. N Engl J Med.

[19] Chapman PB, Hauschild A, Robert C (2011). Improved survival with vemurafenib in melanoma with BRAF V600E mutation. N Engl J Med.

[20] Curtin JA, Busam K, Pinkel D, Bastian BC (2006). Somatic activation of KIT in distinct subtypes of melanoma. J Clin Oncol.

[21] Hodi FS, Friedlander P, Corless CL (2008). Major response to imatinib mesylate in KIT-mutated melanoma. J Clin Oncol.

